# Has Madagascar Lost Its Exceptional Leptospirosis Free-Like Status?

**DOI:** 10.1371/journal.pone.0122683

**Published:** 2015-04-14

**Authors:** Maherisoa Ratsitorahina, Soanandrasana Rahelinirina, Alain Michault, Minoarisoa Rajerison, Soatiana Rajatonirina, Vincent Richard, 2011 Surveillance Workshop group

**Affiliations:** 1 Epidemiology Unit, Pasteur Institute of Madagascar, BP 1274, Antananarivo, Madagascar; 2 Plague Unit, Pasteur Institute of Madagascar, BP 1274, Antananarivo, Madagascar; 3 Biology, Virology and Parasitology Units, Centre Hospitalier Universitaire (CHU Sud), St. Pierre, Reunion Island, France; 4 Epidemiology Unit, Pasteur Institute of Dakar, BP 220, Dakar, Senegal; University of Kentucky College of Medicine, UNITED STATES

## Abstract

**Background:**

Leptospirosis is a widespread but underreported cause of morbidity and mortality. It has rarely been reported in either humans or animals in Madagascar.

**Methods:**

We conducted a cross-sectional survey of the inhabitants in Moramanga, Madagascar, in June 2011, to estimate the prevalence of human infection using the microscopic agglutination test (MAT). This activity was carried out as part of a workshop implemented by the Pasteur Institute of Madagascar, focusing on surveillance with a one week field study and targeting the health staff of the district level.

**Results:**

In total, we sampled 678 inhabitants from 263 households. The sex ratio (M/F) was 0.65 and the mean age 26.7 years. We obtained a value of 2.9% for the first recorded seroprevalence of this disease in the human community of Moramanga. Questionnaire responses revealed frequent contacts between humans and rodents in Moramanga. However, activities involving cattle were identified as a risk factor significantly associated with seropositivity (OR=3).

**Conclusion:**

Leptospirosis remains a neglected disease in Madagascar. This study highlights the need to quantify the public health impact of this neglected disease in a more large scale, in all the country and to establish point-of-care laboratories in remote areas.

## Introduction

Leptospirosis is a worldwide zoonotic infection for which incidence is highest in tropical regions [1,2], constituting a major public health problem in developing countries. Humans are usually infected by contact with urine of an infected host, contaminated drinking water or soil, or infected animal tissue. Notorious reservoirs are rodents, but reservoirs include a variety of wild and domestic animals, livestock, and insectivores.

Leptospira are conventionally divided into two species, the pathogenic *Leptospira interrogans* and the saprophytic *Leptospira biflexa*. More than 60 serovars have been described in the latter and over 250 serovars in 25 serogroups are recognized in *L*.*interrogans*.

It has long been a matter of concern for public health in the southwestern Indian Ocean [3–5]. On the islands close to Madagascar, incidence ranges of 5/100,000 for Reunion Island, 9/100,000 for Mayotte and 101/100,000 for the Seychelles have been reported [5]. The environmental and socioeconomic conditions of Madagascar, with its tropical climate, rice and sugar cane agriculture, livestock farming, slums and the presence of the notorious commensal rodents *Rattus norvegicus* (brown rat) and *R*. *rattus* (black rat), would be expected to favor leptospirosis transmission [6]. However, despite the similarity between conditions on Madagascar and on other nearby islands, the disease has rarely been reported in either humans or animals on Madagascar, where diagnosis is based solely on indirect evidence obtained through antibody detection. A study carried out in the Toliara district in 1968 detected both human and animal leptospirosis. Silverie *et al*. reported that 51% of patients with clinically suspected disease were seropositive for serogroups Tarassovi, Grippotyphosa, Hebdomadis and Australis, and that the seroprevalence of *Leptospira* was 46% in cattle and 8% in pigs [7]. However, subsequent efforts to detect the infection failed to confirm these results. In a survey conducted in Antananarivo, on 2646 serum samples from subjects with no symptoms suggestive of leptospirosis, agglutinating antibodies against the Icterohaemorrhagiae, Grippotyphosa and Canicola serogroups were found in only five samples [8]. In a subsequent study, 105 workers with occupational exposure to *Leptospira* underwent serological screening; antibodies were found in only one worker, and their titer was low [9].

Animal surveys have reported an absence of seropositivity in dogs, sheep, donkeys, horses, cattle and pigs from other sites [10]. No pathogenic strains were obtained from bacteriological cultures of kidney samples from 55 *R*. *rattus* and 50 *Pteropus rufus* (Madagascan flying fox) collected at Marovitsika-Anjiro (100 km north of Antananarivo)[8]. A more recent PCR-based study detected no kidney carriage in 115 rats, 50 zebu cattle and 13 pigs from various sites [9].

The first direct evidence of widespread leptospiral carriage in small mammals in Madagascar was obtained in 2009 [11]. Rates of infection, calculated from the frequencies of positive PCR results, were highest in Moramanga (54%), Toliara (48%) and Mahajanga (47.4%). The 10 isolates obtained from nine rats were all identified as species L. *interrogans* serogroup Canicola serovar Kuwait and all had identical partial *rrs* and *secY* sequences[11]. However, the geographic restriction of seropositivity in *Rattus* species to the Moramanga district remains unexplained. The epidemiological features of leptospirosis in this setting require further investigation. We therefore carried out a survey in Moramanga in 2011, to estimate the prevalence of specific antibodies in humans.

## Methods

### Setting

This study was conducted in Moramanga, a low-income semi-urban area, on the central eastern region of Madagascar. This area was selected because it was here that the DNA carriage in *Rattus* species (54%) was highest in 2009 [11]. The survey was carried out over the course of one week in June 2011, at the start of the cold season, as part of a workshop for the training of health district staff in the use of surveillance tools.

### Sampling

A cross-sectional study was conducted between May 31^st^ 2011 and June 4^th^ 2011, in all the neighborhoods of Moramanga. Households were selected at random for study in each neighborhood. All family members were included in the study, for each household.

### Data collection

A predesigned, semi-structured and validated questionnaire was used for data collection during house-to-house visits. Participants were asked to provide sociodemographic information, and information about the nature of their employment, potential contact with animals whilst at work, the type of contact with animals, the presence of rats at home and in the workplace, types of contact and environmental factors (Table 1).

**Table 1:** Characteristics of the study population in Moramanga, 2011, and risk factors for leptospirosis.

	All *N* = 813		Sampled *N* = 678		Positive *N* = 20		
	*n*	(%)	*n*	(%)	*n*	(%)	*p*
**Sex**							
male	334	(41)	266	(39)	7	(35)	0.82
female	477	(59)	410	(61)	13	(65)	
**Age**							
median	22		23		17		0.17
range	[0.5–87]		[0.5–87]		[3–55]		
**Presence of rats in the household**	691	(85)	585	(86)	16	(80)	0.33
Noise in the ceiling	492	(72)	414	(71)	10	(63)	0.45
Food eaten	582	(84)	499	(85)	14	(87)	0.99
Rat feces in the rooms	462	(67)	404	(70)	12	(75)	0.78
**Water used for washing**							
River or pond	174	(21)	42	(21)	5	(25)	0.58
Tap	345	(43)	297	(44)	9	(45)	0.99
Well	343	(42)	285	(42)	9	(45)	0.83
Other (specified)							
**Drinking water**							
River or pond	35	(4)	30	(4)	1	(5)	0.60
Tap	390	(48)	332	(49)	9	(45)	0.82
Well	394	(49)	320	(47)	10	(50)	0.82
Other (specified)							
**Work setting**							
Office	527	(65)	448	(66)	11	(55)	0.33
Workshop	17	(2)	16	(2)	0	(0)	-
In the fields	114	(14)	98	(15)	3	(15)	0.99
Outdoors, but not in the fields	95	(12)	75	(11)	4	(20)	0.26
Shed or barn	12	(1)	9	(1)	0	(0)	-
**Presence of rats in the workplace**	486	(60)	418	(62)	8	(40)	0.07
Noise in the ceiling	312	(66)	269	(66)	3	(38)	0.15
Food eaten	345	(72)	296	(72)	6	(75)	0.99
Rat feces in the rooms	278	(58)	252	(62)	3	(38)	0.21
**Contact with animals**	727	(90)	601	(90)	19	(95)	0.71
Rats	552	(96)	453	(95)	14	(93)	0.51
Dogs	230	(32)	186	(31)	8	(42)	0.31
Cats	229	(31)	194	(32)	6	(32)	0.99
Pigs	156	(21)	133	(22)	1	(5)	0.09
Cattle	89	(12)	75	(12)	6	(31)	**0.02**
Bats	26	(4)	25	(4)	0	(0)	-
**Leisure activities**							
Fishing in the river	157	(19)	141	(21)	2	(10)	0.39
Swimming in the river	262	(32)	226	(33)	6	(30)	0.81
Hunting	20	(2)	10	(1)	0	(0)	-
**Last febrile illness**							
Currently	54	(7)	46	(7)	2	(10)	0.63
Last month	107	(13)	93	(14)	3	(15)	0.75
Last 12 months	45	(6)	39	(6)	2	(10)	0.32
More than one year	186	(23)	163	(24)	2	(10)	0.18
**History of travel**							
During the last week	20	(2)	14	(2)	0	(0)	-
Last month	32	(4)	27	(4)	1	(5)	0.56
Last 12 months	43	(5)	30	(4)	1	(5)	0.60
More than one year	104	(13)	84	(12)	2	(10)	0.99

### Microscopic agglutination tests (MAT)

We performed the standard MAT reference test as described by Faine *et al*. [12], using a battery of 15 serovars of *Leptospira* spp. as antigens. All strains were maintained in liquid Ellinghausen-McCullough-Johnson-Harris medium (Biorad 55954) at 28°C for one week before testing.

MAT titers are reported as the reciprocal of the largest dilution resulting in the agglutination of at least 50% of the live bacterial antigen, and titers of at least 1:100 were considered positive [13]. The strains were added to serially diluted serum samples in 96-well flat-bottom microtiter plates, which were then incubated at room temperature for two hours. Agglutination was then evaluated by dark-field microscopy at 10 or 20X magnification. The *Leptospira* species included in the antigen panel are listed in Table 2. Serovars for which 50% agglutination was achieved with the highest sample dilution were considered to be probable predominant serovars. Serum samples were considered to be MAT-negative if no agglutination was detected.

**Table 2:** *Leptospira* strains used in this study.

Species	Serogroup	Serovar	Strain
*L*.*interrogans*	Australis	Australis	Ballico
*L*.*interrogans*	Autumnalis	Autumnalis	Akiyami A
*L*.*interrogans*	Canicola	Canicola	Hond Utrecht IV
*L*.*borgpetersenii*	Ballum	Castellonis	Castellon 3
*L*.*borgpetersenii*	Sejroe	Hardjobovis	Sponselee
*L*. *interrogans*	Ictero	Copenhageni	Wijnberg
*L*. *noguchii*	Panama	Panama	CZ 214 K
*L*.*biflexa*	Semaranga	Patoc	Patoc I
*L*. *interrogans*	Pomona	Pomona	Pomona
*L*. *interrogans*	Pyrogenes	Pyrogenes	Salinem
*L*.*borgpetersenii*	Sejroe	Sejroe	M84
*L*.*borgpetersenii*	Tarassovi	Tarassovi	Perepelistin
*L*.*interrogans*	Canicola	kuwait	TOA 25
*L*.*interrogans*	Canicola	kuwait	TOL 17
*L*.*interrogans*	Canicola	kuwait	TOL 55

### Statistical analysis

Data analyses were performed with R software[14]. For all statistical tests, a *p*-value less than 0.05 was considered to denote statistical significance. Qualitative variables are expressed as percentages. Groups were compared in Fisher’s exact tests for categorical variables and quantitative variable were compared in nonparametric tests (Kruskal-Wallis tests).

### Ethics statement

Written informed consent was obtained from each adult participant and from the parent or guardian of each child before enrollment. The study was approved by the National Ethics Committee of the Ministry of Health of Madagascar.

## Results

In total, 263 households and 813 inhabitants participated in the study. Blood samples were obtained from 678 subjects. Serum was obtained from these samples and subjected to the microscopic agglutination test (MAT) for antibodies. The median number of inhabitants per household was 3 (range: 1–10). In total 477 of the subjects studied (59%) were female, and 334 (41%) were male. The mean age of the study subjects was 25.7 years (95% CI: [24.6–27.1]) and the median age was 22 years (range: 6 months to 87 years).

In total, 410 (61%) of the subjects from whom blood samples were collected were female and 266 (39%) were male. The mean age of these subjects was 26.7 years (95% CI: [25.4–28.1]) and their median age was 23 years (range: 6 months to 86 years). The characteristics of the total study population did not differ significantly from those of the population of subjects from whom blood samples were obtained (Table 1).

MAT results were positive for 20 of the 678 subjects from whom samples were collected; seroprevalence was therefore estimated at 2.9% (95%CI: [1.9%-4.7%]). MAT detected antibodies against the Icterohaemorrhagiae (*n* = 16), Panama (*n* = 3) and Canicola (*n* = 2) serogroups. Antibodies against both the Icterohaemorrhagiae and Canicola serogroups were found in one individual.

Contact with cattle was the only risk factor identified in univariate analysis (OR: 3.4 95% CI: [1.03–10.03], *p*-value = 0.02; Table 1).

## Discussion

The primary aim of this survey was to demonstrate the existence of exposure to leptospirosis in the human population of Moramanga, as suggested by the recent finding of an estimated DNA carriage of 54% in the mammalian population of the area [11]. The prevalence of leptospirosis in the inhabitants of Moramanga was estimated at 2.9%. Based on our recent findings, Madagascar seems to have lost its exceptionally low leptospirosis prevalence status among the islands of the Indian Ocean. The estimated prevalence of leptospirosis was higher than that for Reunion Island, which was estimated at about 1.1% in 1991 [15] and at less than 1% in 2006 [3]. However, the prevalence of leptospirosis in Moramanga seems to be similar to that is some areas of Reunion Island with high rainfall. Duval *et al*. [15] reported differences in prevalence as a function of rainfall, from 0.7% on the west coast to 3.1% in south-eastern areas. However, the prevalence of leptospirosis appeared to be lower in Moramanga than in the Union of Comoros. A recent study reported estimated prevalences of 3.4% in Anjouan, 4.2% in Grande-Comore and 10.3% in Mohéli [16].

Previous investigations in Madagascar failed to detect leptospirosis in humans. Lhuiller *et al*. [8] reported a low seroprevalence in the inhabitants of Antananarivo. However, in 1968, Silverie *et al*. reported a prevalence of 51% in selected patients [17] with clinical symptoms, but this result cannot be compared with that reported here since this new study was a cross-sectional community-based study. Furthermore, these new results are not representative of all the country because limited to the population in Moramanga.

The prevalence of the different *Leptospira* serogroups in human populations depends strongly on the local reservoir hosts and the strains they carry [18]. The only risk factor identified in this study was contact with cattle. Bovine leptospirosis has already been suspected in Madagascar [19], but the renal carriage of *Leptospira* in cattle has never been documented [9]. The ecology of leptospirosis in this region of Madagascar, including the range and importance of various reservoir animals, is, therefore, probably not completely understood. The rat is recognized as the major reservoir host for the bacterium, but recent data from Reunion Island indicate that almost all mammals can act as a source of contamination. Pathogenic *Leptospira* spp. (*L*. *borgpetersenii*, *L*. *interrogans*) were recently found in bats in Madagascar and the Union of Comoros [20]. A better knowledge of animal leptospirosis is therefore essential, to improve assessments of the risk to humans. The study area for this investigation was therefore selected on the basis of results for surveillance in mammals [11].

Acute leptospirosis has never been reported in Madagascar. In humans, clinical leptospirosis has diverse manifestations, but it generally causes a febrile illness that is often difficult to distinguish from other acute influenza-like fevers, such as dengue, influenza, chikungunya and malaria during its early stages [1]. A lack of appropriate laboratory facilities is the principal reason for the failure to diagnose leptospirosis in Madagascar, highlighting the need for point-of-care laboratories in remote areas [21].

Madagascar is a large island and our cross-sectional study was restricted to Moramanga district. This is undoubtedly a limitation for conclusion about the all country leptospirosis status but is an important first step to go further in the description of the epidemiological profile of the country.

## Conclusion

Information about leptospirosis in Madagascar remains scare. Risk assessment should take into account not only the diagnosis of incident cases in humans, but improvements in our knowledge of animal risks and the correspondence between human seroprevalence data and different climate areas and seasons.
